# A Prolonged Ventricular Standstill Episode During a Total Hip Replacement—A Case Report

**DOI:** 10.1155/cric/5161799

**Published:** 2026-07-29

**Authors:** Katelyn Eisendrath, Michael Carter, Dakota Robertson

**Affiliations:** ^1^ Department of Internal Medicine, Johnston Memorial Hospital, Abingdon, Virginia, USA

**Keywords:** case report, inflammation, vasovagal response, ventricular standstill

## Abstract

**Background:**

Ventricular standstill, defined as the complete cessation of ventricular contraction, is a rare and sometimes fatal phenomenon. Few cases have noted patients to be hemodynamically stable and asymptomatic during the event, and no cases have identified ventricular standstill to occur during surgery.

**Case Summary:**

A 63‐year‐old male presented after an elective right total hip replacement, with telemetry revealing a 20‐s absence of ventricular contractility during surgery. This is consistent with ventricular standstill. The patient was asymptomatic and hemodynamically stable before, during, and after the event.

**Conclusions:**

This case is rare due to the prolonged episode of ventricular standstill that occurred during surgery and the fact that the patient remained hemodynamically stable. Possible pathophysiological mechanisms of action of this phenomenon include both a vasovagal and inflammatory response, especially in a surgical setting with anesthesia.

## 1. Introduction

Ventricular standstill is a rare phenomenon that presents with P waves without correlating QRS complexes on an electrocardiogram [1, 2]. It has been recognized as a potentially fatal phenomenon, and few case reports have identified it often occurring after surgery [2], with profuse vomiting [3], or in the setting of Takotsubo′s cardiomyopathy [1]. Many patients, but not all, present with syncope and altered mental status due to a significant reduction in cardiac output secondary to lack of ventricular depolarization [4]. As seen in the case report outlined below, some patients can be hemodynamically stable or asymptomatic during the event.

## 2. Case Report

**Figure 1:**
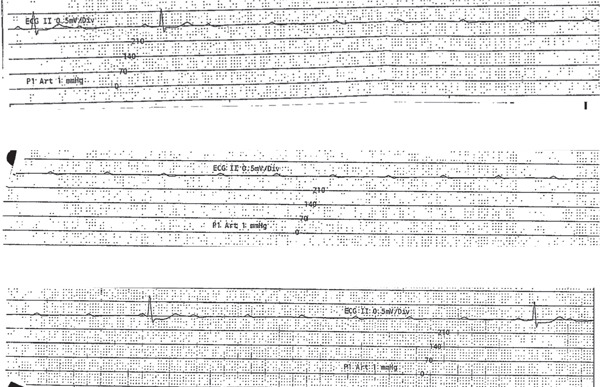
Telemetry strips revealing a 20‐second ventricular standstill event.

A 63‐year‐old male, with a past medical history of hypertension, hyperlipidemia, osteoarthritis, and chronic obstructive pulmonary disease, was admitted to the hospital primarily for an episode of prolonged ventricular standstill. He had an outpatient, scheduled, elective right total hip replacement on July 15, 2025. During surgery, the patient had a 20‐second cardiac pause on telemetry, as shown in Figure 1, at 10:27 in the morning. Throughout this ventricular standstill episode, he remained hemodynamically stable. A 12‐lead electrocardiogram was later completed at 12:54 in the afternoon and revealed sinus rhythm with first‐degree atrioventricular (AV) block. In the postanesthesia care unit after surgery, the patient was conversant and alert. He denied any recent episodes of syncope, near‐syncope, chest pain, shortness of breath, and any personal or family history of coronary artery disease.

He was closely monitored on telemetry for the remainder of his hospital stay, and cardiology was consulted. Two repeat electrocardiograms completed on July 16, 2025, revealed normal sinus rhythm with no AV block. Transthoracic echocardiography completed on July 16, 2025, revealed a left ventricular ejection fraction of 60%, mild concentric left ventricular hypertrophy, and no significant valvular abnormalities. Cardiology recommended a 14‐day Holter monitor with an outpatient follow‐up appointment, and the patient was discharged. The extended‐wear Holter monitor was conducted from July 16, 2025, to July 30, 2025. It noted a sinus rhythm of 48 beats per minute with a maximum of 129 beats per minute, an average heart rate of 69 beats per minute, modest bradycardia during presumably awake hours, no pauses, no AV block, and no patient triggers.

This patient later presented to his outpatient follow‐up appointment on September 29, 2025. He mentioned that he does sometimes experience a vasovagal response with injections and needles. Due to no signs or symptoms of underlying conduction disease, the cardiology team attributed this episode of ventricular standstill to either adduction of anesthesia or a vasovagal response during surgery.

## 3. Discussion

Ventricular standstill occurs when the ventricles cease to contract, and cardiac output is temporarily negligible [1, 2]. The pathophysiological mechanism of ventricular standstill has yet to be elucidated, but both a vasovagal and inflammatory response have been proposed to be possible mechanisms of action.

A vasovagal response is one in which the vagus nerve is overstimulated, leading to systemic vasodilation, bradycardia, and AV block [3]. When the AV node is inhibited, electrical current to the ventricles is reduced [3]. This can create an arrhythmia, one of which is ventricular standstill [3]. Few case reports have reported this association between AV blocks and dysrhythmias, specifically ventricular standstill [3–5].

With regard to inflammatory responses, current research suspects that systemic inflammation increases circulating inflammatory cytokines. This subsequently alters cardiomyocyte ion channel function, leading to an increased cardiomyocyte action potential duration [6]. A simulated bench research study further supports this conjecture that specific inflammatory cytokines, including tumor necrosis factor‐alpha, interleukin‐1 beta, and interleukin‐6, do prolong action potential duration [6]. It also further identified that these inflammatory cytokines can both enhance action potential repolarization and reduce ventricular tissue adaptability to tachycardia [6]. Altogether, these pathophysiological changes can lead to ventricular arrhythmias, such as ventricular standstill [6].

It can be speculated that the patient in our case report likely had both inflammatory and vasovagal responses due to the stress of surgery and anesthesia. Furthermore, a case report and literature review published in 2024 identified 35 cases of ventricular standstill from 2000 to 2024 [7]. This article found that 57% of ventricular standstill cases were noted to be female patients, 20% of cases were attributed to myocarditis (consistent with the inflammatory response pathway), 20% of cases were attributed to increased vagal tone, and only two patients (8%) were asymptomatic at the time of diagnosis [7]. This truly highlights how rare our case of ventricular standstill is, as the patient was male and hemodynamically stable during the event. He also did have a first‐degree AV block, supporting the association between AV block and dysrhythmias. Furthermore, after a thorough literature review, no case reports have ever identified ventricular standstill occurring during surgery. Future research should continue to focus on the pathophysiological mechanisms of this phenomenon, especially during surgery.

## 4. Limitations

The patient′s episode of ventricular standstill occurred during surgery. Unfortunately, the start and stop times of the right total hip replacement were not documented in either the surgeon′s note or the nurse anesthetist′s note. It is not possible to determine at what point during the surgery the ventricular standstill episode occurred.

## Funding

No funding was received for this manuscript.

## Consent

The patient provided consent for this case to be published in a journal, in compliance with COPE guidelines.

## Conflicts of Interest

The authors declare no conflicts of interest.

## Data Availability

The data that support the findings of this study are available from the corresponding author upon reasonable request.
